# Quantifying CO_2_ Removal at Enhanced Weathering Sites: a Multiproxy Approach

**DOI:** 10.1021/acs.est.3c03757

**Published:** 2023-06-21

**Authors:** William J. Knapp, Emily I. Stevenson, Phil Renforth, Philippa L. Ascough, Alasdair C. G. Knight, Luke Bridgestock, Michael J. Bickle, Yongjie Lin, Alex L. Riley, William M. Mayes, Edward T. Tipper

**Affiliations:** † Department of Earth Sciences, University of Cambridge, Cambridge CB2 3EQ, United Kingdom; ‡ Research Centre for Carbon Solutions, Heriot-Watt University, Edinburgh EH14 4AS, United Kingdom; § NEIF Radiocarbon Laboratory, Scottish Universities Environmental Research Centre, Glasgow G75 0QF, United Kingdom; ∥ School of Earth and Environmental Sciences, University of St. Andrews, St. Andrews KY16 9TS, United Kingdom; ⊥ MNR Key Laboratory of Saline Lake Resources and Environments, Institute of Mineral Resources, Chinese Academy of Geological Sciences, Beijing 100037, China; # School of Environmental Sciences, University of Hull, Hull HU6 7RX, United Kingdom

**Keywords:** Radiocarbon, Carbon dioxide removal, Mineralisation, Isotopic tracers, Monitoring

## Abstract

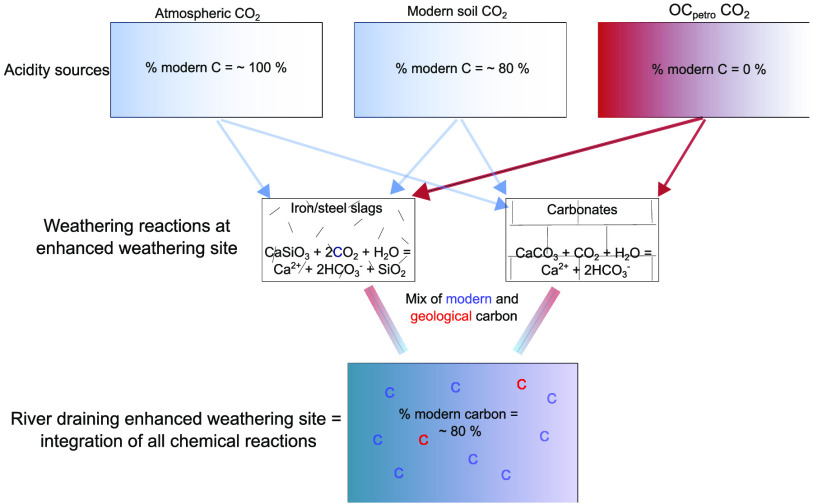

Enhanced weathering is a carbon dioxide (CO_2_) mitigation strategy that promises large scale atmospheric CO_2_ removal. The main challenge associated with enhanced weathering is monitoring, reporting, and verifying (MRV) the amount of carbon removed as a result of enhanced weathering reactions. Here, we study a CO_2_ mineralization site in Consett, Co. Durham, UK, where steel slags have been weathered in a landscaped deposit for over 40 years. We provide new radiocarbon, δ^13^C, ^87^Sr/^86^Sr, and major element data in waters, calcite precipitates, and soils to quantify the rate of carbon removal. We demonstrate that measuring the radiocarbon activity of CaCO_3_ deposited in waters draining the slag deposit provides a robust constraint on the carbon source being sequestered (80% from the atmosphere, 2σ = 8%) and use downstream alkalinity measurements to determine the proportion of carbon exported to the ocean. The main phases dissolving in the slag are hydroxide minerals (e.g., portlandite) with minor contributions (<3%) from silicate minerals. We propose a novel method for quantifying carbon removal rates at enhanced weathering sites, which is a function of the radiocarbon-apportioned sources of carbon being sequestered, and the proportion of carbon being exported from the catchment to the oceans.

## Abstract 2

Limited research exists on quantifying carbon removal rates at field scale enhanced weathering sites. We present an initial framework for quantifying carbon removal rates, which minimizes uncertainties.

## Introduction

Enhanced weathering is a carbon removal strategy aimed at removing carbon dioxide (CO_2_) from the atmosphere. Typically, this involves the spreading of silicate^1−3^ and carbonate^4−6^ rock powders on a variety of land types (e.g., arable, forested). The powders may be sourced from naturally occurring minerals (e.g., wollastonite, calcite) or derived from industrial activities, e.g., mine tailings or blast furnace slags (hereafter referred to as slag deposits).^7^ These rock powders react with atmospheric CO_2_, converting gaseous CO_2_ into aqueous bicarbonate or carbonate ions (HCO_3_^–^, CO_2–_^3^ respectively). The carbon species in solution are either mineralized on land or transported via rivers to the ocean, where they may remain in solution for long time periods (10 000 years^8^) or are precipitated as calcium carbonate (CaCO_3_). Effectively, enhanced weathering increases the rate of natural rock weathering processes, which on long time scales regulate atmospheric [CO_2_].^9,10^ Iron and steel making slags derived from the steel industry are of particular relevance for carbon removal efforts, as the production of 1 tonne of steel results in the emission of 1.8 tonnes of CO_2_. Due to the global demand for steel, steel production is responsible for 7% of global CO_2_ emissions.^11^ Steel demand is expected to increase, potentially 2-fold, by 2050,^12^ making the steel industry a priority sector for decarbonization, a process that can be facilitated by the enhanced weathering of iron/steel-making slags.

Analyzing river (or groundwater) chemistry from catchments undergoing enhanced weathering trials provides an ideal method to quantify the rate of atmospheric carbon removal. This is because river chemistry integrates the suite of water–rock interactions occurring in a given catchment, encompassing mineral dissolution, but also secondary processes (e.g., calcite precipitation, clay formation), which can impact carbon removal efforts.^5^

Studying dissolved inorganic carbon (DIC), major elements, and isotopes in rivers allows quantification of key parameters for calculating carbon fluxes such as (i) the proportion of carbon derived from the modern atmosphere (i.e., active sequestration of modern atmospheric CO_2_); (ii) the proportion of solutes derived from mineral dissolution reactions that are consequential for carbon sequestration; and (iii) the amount of DIC exported to the ocean. Indeed, monitoring, reporting, and verifying (MRV) enhanced weathering has been the focus of discussion in recent years,^13,14^ particularly because of the potential importance of enhanced weathering in emerging voluntary carbon markets.^15^ Previous studies have suggested that using downstream electrical conductivity and total alkalinity are suitable measures for quantifying carbon removal rates by enhanced weathering.^13^ Although rapid and cost-effective, these methods do not partition alkalinity into modern atmospheric carbon and geological carbon. For instance, the dissolution of carbonate rock results in the addition of 2 mol of alkalinity to a solution, one that is derived from modern atmospheric CO_2_ and a second derived from the carbonate rock, which is a geological source. Therefore, carbonate dissolution creates a mixture of modern and geological CO_2_. Measuring only alkalinity or conductivity does not apportion these sources of carbon, which could lead to inaccurate carbon removal estimates. If carbonate phases occur in small quantities in an enhanced rock weathering (ERW) feedstock, making them particularly hard to detect, then it is easy for carbon removal estimates to become overinflated. A similar argument can be made for the oxidation of petrogenic organic carbon (OC_petro_),^16,17^ which can provide a geological acidity source for mineral acid hydrolysis.^18−20^

Here, we present geochemical measurements of streamwater and associated authigenic CaCO_3_ precipitates from a well studied CO_2_ mineralization site, the Consett steel works in Co. Durham, UK. Enhanced weathering at Consett can be considered incidental, as the alkaline, silicate-based steel-slag material at this site has been weathered since the steel works opened over 120 years ago. The Consett site differs from typical ERW sites where a basalt powder is spread on agricultural fields at an application rate of ∼40 t ha^–1^ y^–1^.^21^ At Consett, 20 Mt of steel slag^22^ was placed over a 120 year period and subsequently weathered. This equates to an application rate of ∼1000 t ha^–1^ y^–1^, given the operational period of the steel works (120 years), the amount of material produced during that time (20 Mt), and the slag deposit area (160 ha). This application rate is greater than typical ERW application rates, but the end result is similar, Stream waters draining both ERW sites and the Consett site comprise a mixture of geological and modern sources of CO_2_, some of which is additional because of spreading reactive minerals within the critical zone.

The problem of conducting rigorous MRV at ERW sites is discussed here, whereby a natural system (Howden Burn) with a given baseline chemistry has been perturbed by the addition of reactive silicate minerals (steel slags). Howden Burn presents very similar challenges that other ERW studies have faced, i.e., partitioning carbon sources^14^ and modeling CO_2_ release as a result of secondary mineral precipitation.^5,23^ Consett benefits from being a well studied^22,24−28^ and constrained field site, where mineral dissolution rates are rapid,^26^ which allows these challenges to be addressed thoroughly.

Weathering of steel slags at Consett has resulted in high-pH waters and widespread authigenic CaCO_3_ precipitation in the Howden Burn, a stream draining the site. Previous studies suggest that between 50 and 99% of the carbon in the precipitated authigenic CaCO_3_ could be derived from the atmosphere.^26^ New data and modeling are presented, providing a much tighter constraint on the fraction of atmospheric CO_2_ that is sequestered in both the authigenic CaCO_3_ and stream waters. The new data and modeling framework aims to provide an accurate assessment of the rate of modern carbon removal from this enhanced weathering site, which will provide reference for the challenges associated with quantifying carbon removal rates from other enhanced weathering sites.

## Materials and Methods

### Site Description

**Figure 1:**
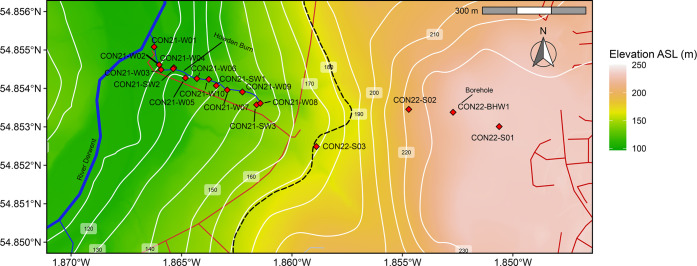
Map of the study area with samples shown (red diamonds). Topographic high to the east of the black dashed line represents the extent of the slag deposit. Roads and access trails to the Howden burn are marked in red. Year of collection precedes sample codes; e.g., CON21 = July 2021 sampling campaign.

Iron and steel were produced in Consett, Co. Durham for over 100 years (beginning in 1840) until production ceased in 1980. During the operational period of the steelworks, >20 million tonnes of slag was produced, now landscaped into several large slag deposits (Figure 1) overlaying consolidated alluvium, glacial till, and Carboniferous rocks, comprising sandstones, limestones, and coal deposits. Consett slag waste is comprised of Ca silicate-rich melilite group minerals such as gehlenite (Ca_2_Al_1_SiO_7_) and akermanite (Ca_2_MgSi_2_O_7_), with trace amounts of portlandite (Ca(OH)_2_).^22^ Silicate minerals react with carbonic acid, formed from CO_2_ dissolving in water in the soil environment, whereas hydroxides dissociate in water, delivering cations and DIC to local waters via the following reaction pathways:
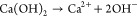
1
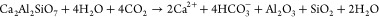
2

In this instance, the portlandite (a minor component in the slag material^22^) in eq 1 removes 1 mol of CO_2_ per mole of Ca^2+^ leached via the following CO_2_ hydroxylation reaction (eq 3), which results in direct carbonation of the portlandite:
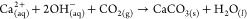
3

The dissolution of gehlenite (a dominant component in the slag material^22^) in eq 2 removes 2 mol of CO_2_ per mole of Ca^2+^ leached (eq 2) and 1 mol of CO_2_ per mole of Ca^2+^ directly carbonated from gehlenite (eq 4):
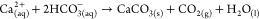
4

Equations 1 and 2 contribute DIC to stream waters at Consett. Additional sources of DIC to stream waters at Consett could include respiration of modern organic matter in soils overlying the slag deposits, oxidation of OC_petro_ (e.g., coal or coke), and dissolution of carbonates from Carboniferous limestones.

Howden Burn (a minor tributary of the River Derwent, Figure 1) emerges at the base of the main slag deposit and is culverted beneath a road and re-emerges once again in a small woodland (Figure 1). Springs emerging from underlying Carboniferous rocks contribute to the total discharge and solute flux at the Howden Burn. Quantification of spring contributions to the total flux is required to quantify carbon removal at the Howden Burn. The main spring input is ∼200 m downstream of the culvert (Figure 1); the two other minor springs are proximal to the culvert and mouth of Howden Burn. There is a significant amount of in situ carbonate precipitation, forming layered authigenic CaCO_3_ deposits on the riverbed (SI Figure 1). Calcite precipitation in waters draining the slag deposit is rapid, estimated to be as great as 100 g of CaCO_3_ m^–2^ day^–1^.^25^ Groundwater from within the slag deposit was sampled from a borehole (Figure 1), providing a water chemistry end member. Howden Burn in Consett represents a unique opportunity to study enhanced weathering as the site is in effect a well-established enhanced weathering and CO_2_ mineralization site, and this is reflected in the streamwater chemistry.

### Sample Collection and Analytical Methods

Samples were collected from Howden Burn, in September 2020, July 2021, and March 2022 (Table 1, Table 2, and Figure 1). Hydrological conditions were consistent during the collection of both sets of water and authigenic CaCO_3_ samples (SI Figure 2), reducing the impact of seasonality. Soil samples were collected using an auger at a depth of 30 cm. Authigenic CaCO_3_ and water samples were taken at ∼50 m intervals within flowing water in the river to ensure the samples were as recent as possible and so external inputs (e.g., from spring and groundwaters) could be assessed. The uppermost layers of calcite were selected from the authigenic CaCO_3_ for radiocarbon analysis and stable isotope (δ^13^C and δ^18^O) analysis, which we assume to have crystallized rapidly within the previous 12 months. To prevent bacterial growth or diagenesis, authigenic CaCO_3_ and soil samples were baked at a low temperature (60 °C) for 5 h, powdered, and stored in airtight bags in the dark. A summary of the authigenic CaCO_3_ and soil samples analyzed is given in Table 2. Water samples were collected in acid-washed containers and filtered immediately after sampling through a 0.2 μm poly(ether sulfone) membrane using a polycarbonate and UPVC filtration unit, into acid washed HDPE bottles. Three aliquots were collected, for cations, isotopes, and anions. Cation and isotope samples were both acidified to pH 2 using distilled HNO_3_. Anion samples were not acidified. A 50 mL unacidified, filtered aliquot of each water sample was titrated in the field, using the gran method^29^ and a Hanna Instruments HI-991301 pH meter (0.01 resolution). Replicate alkalinity measurements reproduced values to within 1% of the original value.

**Table 1:** Chemistry Data for Stream Water (W), Spring Water (SW), and Borehole Water (BHW) Samples Taken from Howden Burn^a^

sample ID	lat, deg	lon, deg	^87^Sr/^86^Sr	^87^Sr/^86^Sr, 2σ ppm	δ^13^C, ‰	pH	alkalinity, μmol L^–1^	Ca, μmolL^–1^	Mg, μmolL^–1^	Sr, μmolL^–1^	Si, μmolL^–1^	Si/Cl, mol/mol	NICB, %
W01	54.855	–1.866	0.7089954	21	–13.24	11.00	864.6	2082.3	92.6	22.9	142.1	0.15	–4.1
W02	54.855	–1.866	0.7089916	20	–13.22	11.08	1418.0	2221.6	95.0	23.6	146.3	0.15	–6.0
W03	54.854	–1.866	0.7089893	7	–16.53	11.09	1374.6	2259.0	90.1	23.9	144.2	0.16	–5.8
W04	54.855	–1.865	0.7089951	26	–14.55	11.26	1743.2	2372.8	90.1	24.7	148.5	0.16	–6.6
SW2	54.854	–1.865	0.7116435	16			2670.5	1082.3	577.7	1.4	161.3	0.13	–3.0
W05	54.854	–1.865	0.7090009	6		11.27	1889.2	2500.7	91.3	25.3	151.7	0.16	–6.9
W06	54.854	–1.864	0.7090158	34	–14.55	11.60	2240.8	2553.9	85.2	25.9	148.5	0.16	–8.2
SW1	54.854	–1.864	0.7097111	20	–17.50	8.21	1940.6	2251.5	451.8	6.4	145.3	0.15	–7.3
W10	54.854	–1.863	0.7089592	21	–16.62	11.62	2606.5	3311.4	34.6	36.4	181.9	0.20	–8.0
W07	54.854	–1.863	0.7089825	16	–15.30	11.73	2894.4	2914.7	30.9	30.8	158.1	0.17	–8.1
W09	54.854	–1.862	0.7089515	8	–15.96	11.78	3297.8	3494.8	34.6	37.1	174.5	0.21	–8.6
SW3	54.854	–1.862	0.7102273	26		8.13	5081.4	3610.0	1247.1	5.3	214.3	0.18	–6.4
W08	54.854	–1.862	0.7089634	15	–16.78	11.90	4098.0	3482.0	30.9	33.9	159.2	0.20	–8.2
BHW1	54.853	–1.850	0.7088532	9		12.00	5025.6	5160.9	2.9	68.3	122.1	0.60	–9.9

a NICB is normalized inorganic charge balance.

**Table 2:** Stable Isotope (δ^13^C and δ^18^O) and Radiocarbon pMC (Percent Modern Carbon) and pTC (Percent Total Carbon) Data for Travertines (T), Soil Carbon (S), and Replicates (R) Sampled in This Study

sample ID	lat, deg	lon, deg	δ^18^O, ‰	δ^13^C, ‰	pMC, %	pMC 1σ, %	pTC, wt %
T02	54.855	–1.866	–9.89	–16.87	86.16	0.39	10.21
T03-R1	54.854	–1.866	–8.91	–16.21	80.86	0.38	10.97
T03-R2	54.854	–1.866	–9.35	–16.30	80.71	0.39	11.25
T03	54.854	–1.866	–9.51	–16.66			
T04	54.855	–1.865	–9.68	–16.86	80.84	0.39	11.32
T05	54.854	–1.865	–9.60	–16.92	83.09	0.40	11.00
T06	54.854	–1.864	–9.03	–16.86	83.80	0.40	11.22
T10	54.854	–1.863	–10.93	–17.09	86.38	0.42	10.83
T07	54.854	–1.863	–9.31	–15.85	73.91	0.36	10.77
T08a	54.854	–1.862	–9.72	–15.89			
T08b	54.854	–1.862	–8.33	–14.06			
S01	54.853	–1.850		–24.83	26.83	0.15	4.94
S02	54.853	–1.854		–26.07	52.86	0.26	4.80
S03	54.852	–1.858		–25.77	41.05	0.21	2.75

Radiogenic Sr isotopes (^87^Sr/^86^Sr) were measured on a Neptune Plus MC-ICP-MS instrument at the University of Cambridge. Sr was separated from the sample matrix using Biorad Micro Bio-Spin columns with Eichrom Sr spec resin.^30^ The Sr fraction was then dried to a salt and dissolved in 2% HNO_3_. Samples were introduced to the plasma via the APEX IR sample introduction system and an ESI 50 μL PFA nebulizer at a concentration of 50 ppb Sr. Samples were run in triplicate, and interferences were corrected for by on-peak zeros by subtracting the blank measurements bracketing the sample measurement. Any additional ^85^Rb in the samples was corrected using an exponential correction. The exponential law was applied to correct for instrument mass fractionation; ^87^Sr/^86^Sr ratios were normalized to ^87^Sr/^86^Sr = 0.1194. Every five samples were bracketed by the NBS 987 standard, which gave a ^87^Sr/^86^Sr of 0.710270 ± 80 ppm (2σ, *n* = 122). IAPSO seawater was processed through column chemistry yielding a ^87^Sr/^86^Sr ratio of 0.709195 ± 24 ppm (2σ, *n* = 5), within uncertainty of the accepted value, 0.709179 ± 8 ppm.^31^

Cation concentrations were measured with an ICP-OES (Agilent 5100) at the University of Cambridge. Concentrations of cations were determined against a synthetic calibration line and checked via standards SPS-SW2 and SLRS6 (National Research Council Canada). Standards were reproduced to better than 5% for elements Ca, Mg, Sr, and Si reproducible to 7%.

Stable metal isotopes, δ^13^C and δ^18^O, were measured at the Godwin lab (University of Cambridge) on a Thermo Delta V Adv. Gasbench^32^ with a precision of 0.1 ‰ (2σ). Authigenic CaCO_3_ and soil samples were prepared for radiocarbon measurement at the Natural Environmental Isotope Facility (NEIF) radiocarbon laboratory at the Scottish Universities Environmental Research Centre (SUERC). Carbonates were etched with weak HCl, and soils were pretreated by acid fumigation. Evolved CO_2_ was cryogenically purified on a vacuum line. Purified CO_2_ was converted to graphite by the Fe/Zn reduction method.^33^ Radiocarbon composition of graphite was then measured by using an accelerator mass spectrometer (AMS) at the SUERC AMS laboratory. Additional method details are in the SI.

## Results and Discussion

### Water Chemistry

**Figure 2:**
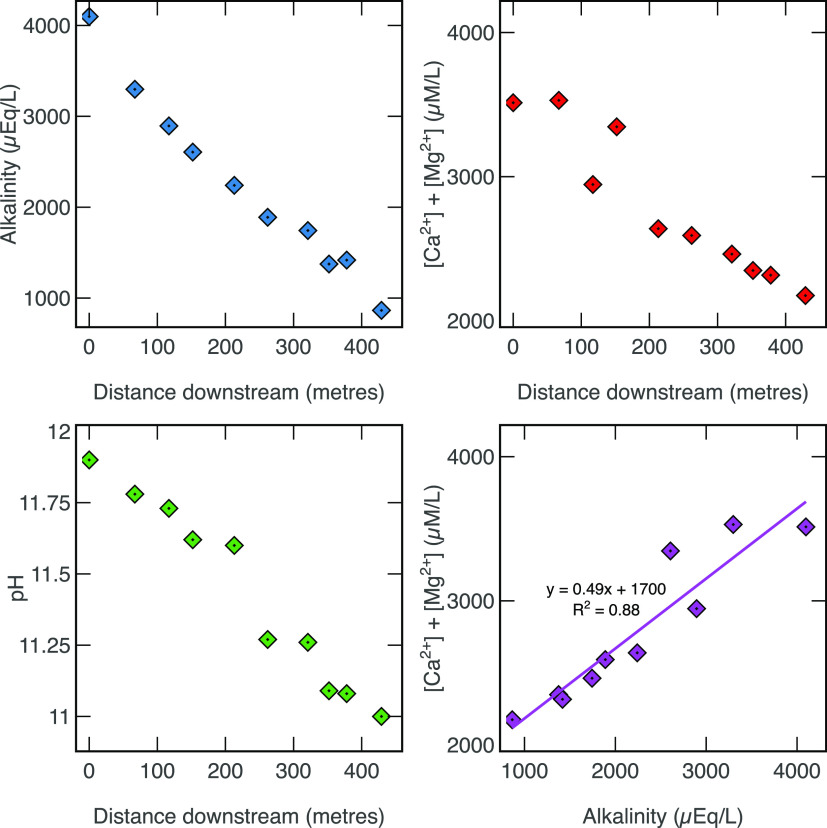
(a) Downstream plot of alkalinity, (b) downstream plot of Ca + Mg, (c) downstream plot of pH, and (d) cross-plot of Ca + Mg and alkalinity. Analytical uncertainty is smaller than the data points.

**Figure 3:**
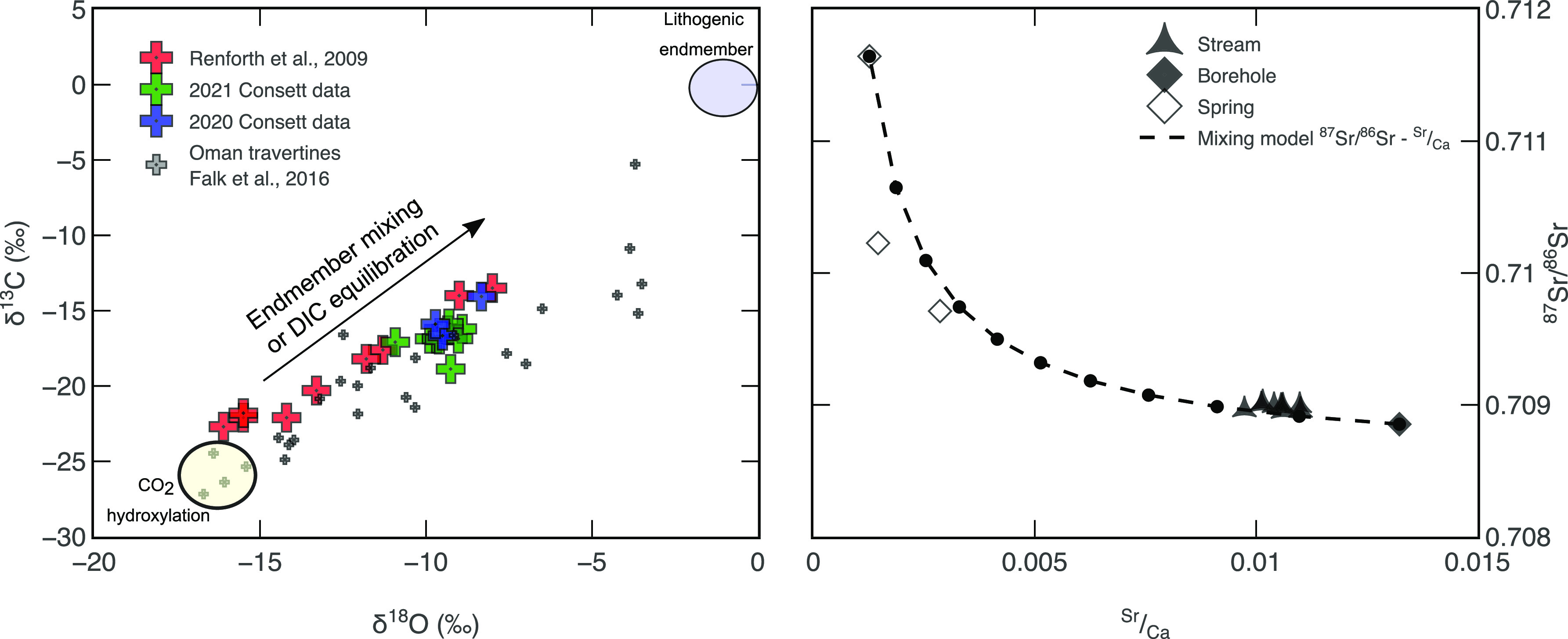
(a) Mixing model in δ^18^O−δ^13^C space between CO_2_-hydroxylation end member after^37^ and lithogenic end member (δ^18^O = 0 ‰, δ^13^C = −2 ‰) after.^26,40^ (b) Mixing model between SW1 and borehole end member, BHW1). Deviation in Sr/Ca in the stream is thought to be a result of carbonate precipitation. We predict that the borehole end member satisfies 80–90% of the streamwater chemical composition.

Alkalinity along Howden Burn decreases linearly with distance downstream from 4098 μeq L^–1^ at the culvert (W08) to 864 μeq ^–1^ at confluence with Derwent (W01), [Ca+Mg] decreases from 3512 μmol L^–1^ to 2174 μmol L^–1^, and pH drops an entire unit from 12.0 to 11.0 over a ∼425 m distance downstream (Figure 2). [Ca+Mg] and alkalinity are well correlated downstream (*R*^2^ = 0.88, Figure 2). Stream water δ^13^C values increase downstream, from −16.78 ‰ to −13.24 ‰ (Table 1), whereas ^87^Sr/^86^Sr is relatively consistent in stream and borehole waters (Table 1), with a mean of 0.708972 (2σ = 88 ppm, *n* = 11). Spring water samples (SW1, SW2, SW3, Table 1) have markedly different ^87^Sr/^86^Sr in comparison to stream and borehole waters, representing a different water provenance. Spring waters have a mean ^87^Sr/^86^Sr of 0.710527 (*n* = 3). Spring and borehole waters represent end-member ^87^Sr/^86^Sr compositions. Because of the lime-based purification process of steel-making, borehole waters draining silicate-based slag material define a carbonate-type end member (∼0.709, Figure 3b),^34^ contrasting spring waters draining the local geology, which define either a mostly silicate-based end member (SW1) or a mixture between carbonate and silicate weathering (SW2 and SW3, ∼0.710, Figure 3b).^9,34,35^ Sr/Ca ratios follow a similar trend to that of ^87^Sr/^86^Sr, being consistent in stream and borehole waters and offset in spring waters (Table 1). Stream and borehole waters have an average Sr/Ca of 0.01, whereas spring waters have an average Sr/Ca an order of magnitude lower, 0.001 (Table 1). All solute data can either be explained by nonconservative CaCO_3_ precipitation as distance downstream increases or conservative mixing between two water end members.

### Authigenic CaCO_3_ and Soil Chemistry

Authigenic CaCO_3_ δ^18^O data show consistent values downstream, with a mean of −9.48 ‰ (Table 2, 2σ = 1.3 ‰, n = 11), and are generally isotopically heavier than previous δ^18^O measurements of authigenic CaCO_3_ from an adjacent, but hydrologically unconnected, site.^27^ The isotopic enrichment observed between the sites is likely induced by differing hydrological conditions. Whereas Howden Burn is fed almost entirely by groundwater derived from the slag deposit, the wetland site previously studied is fed by a mixture of streamwater draining Carboniferous coal measures and groundwater draining the slag deposit.^27^ Authigenic CaCO_3_ δ^13^C is similarly consistent, with a mean of −16.32 ‰ (Table 2, 2σ = 1.7 ‰, n= 11), and falls within previous estimates of δ^13^C in authigenic CaCO_3_.^27^ Radiocarbon data are also consistent between samples, with a mean percent modern carbon (pMC) of 82% (Table 2, 2σ = 8%, *n* = 8). The mean wt % carbon in the authigenic CaCO_3_ samples is 11%, suggesting the samples are close to pure CaCO_3_. This is corroborated by XRD data (*n* = 3), which show the authigenic CaCO_3_ to be almost entirely calcite with trace montmorillonite (SI Figure 3). Organic carbon in soils, collected at Howden Burn, shows δ^13^C values typical of organic matter, with a mean δ^13^C of −25.56 ‰ (Table 2, *n* = 3). Radiocarbon measurements of soil carbon have a mean pMC of 40% (2σ = 88%, Table 2).

### Estimating the Proportion of Atmospheric CO_2_ Sequestered at Howden Burn

Accurately quantifying atmospheric carbon removal rates from enhanced weathering and mineralization requires knowledge of the proportion of atmospheric CO_2_ being sequestered. Previous attempts at quantifying the fraction of modern carbon in authigenic CaCO_3_ at Howden Burn, using stable metal isotopes (δ^13^C and δ^18^O), are limited by large propagated uncertainties (∼50%, Figure 3a).^26,27,36^ This is because δ^13^C and δ^18^O measurements in authigenic CaCO_3_ are unlikely to be solely influenced by conservative mixing between two chemically distinct end members, in this case a geological source of carbon that is lithogenic, and a modern source of carbon, i.e., hydroxylated CO_2_ (Figure 3a).^26,36,37^

Deviations from conservative mixing in δ^13^C and δ^18^O space are also seen in authigenic CaCO_3_ derived from dissolution of the Oman ophiolite, which produces hyper-alkaline solutions > pH 11 similar to those at Howden Burn (Figure 3a).^37^ Nonconservative processes such as the partial equilibration of DIC in stream waters (Figure 3a)^37^ as well as conservative mixing were invoked to explain the Oman data.^37^ Partial DIC equilibration is the result of CO_2_ hydroxylation reactions preferentially incorporating the lighter stable carbon and oxygen isotopes (^12^C and ^18^O) into solution, which are then rapidly precipitated as CaCO_3_ before equilibrating with waters.^36,37^ Because of the potential influence of nonconservative processes, measuring δ^13^C and δ^18^O in authigenic CaCO_3_ precipitated from high-pH waters is unlikely to provide a unique solution, from which the source of carbon can be accurately estimated.

Spring water and borehole provenance was defined by direct measurement of ^87^Sr/^86^Sr and Sr/Ca ratios (Figure 3b). This allows quantification of the extent of conservative mixing, independent of nonconservative behavior associated with the precipitation of authigenic CaCO_3_. Stream water chemistry is dominated by contributions from the borehole, 80–90%, implying that the dissolution of slag deposit minerals dominates the chemistry of Howden Burn, corroborating long-term chemical data from waters draining the slag deposit.^25^ Since the majority of stream solutes are derived from the slag heap sampled by the borehole (Figure 3b), where eqs 1 and 2 are thought to dominate the alkalinity budget, it follows that >80% of the carbon in waters, hence authigenic CaCO_3_, should be derived from the modern atmosphere. However, this comes with the caveat that each chemical end member has an assumed dissolution reaction associated with it; e.g., borehole water represents modern carbon; spring waters represent geological carbon. This assumption may not be entirely true, as spring waters are shown here to span between carbonate and silicate weathering (Figure 3b), which would supply a mix of modern and geological carbon.

The radiocarbon composition of the authigenic CaCO_3_ provides an excellent constraint on the source of the carbon being mineralized at Howden Burn, with all samples producing similar pMC values (Table 2). This is because the normalization of measured ^14^C/^12^C to δ^13^C corrects for the effects of isotope fractionation during exchange between DIC pools (e.g., CO_2_ invasion, carbonate precipitation, and CO_2_ degassing).^38^ Measuring the radiocarbon content of authigenic CaCO_3_ provides the capability to simplify all of the potential sources of carbon into a binary mixture of radiocarbon, dead carbon, or modern carbon.^39^

**Figure 4:**
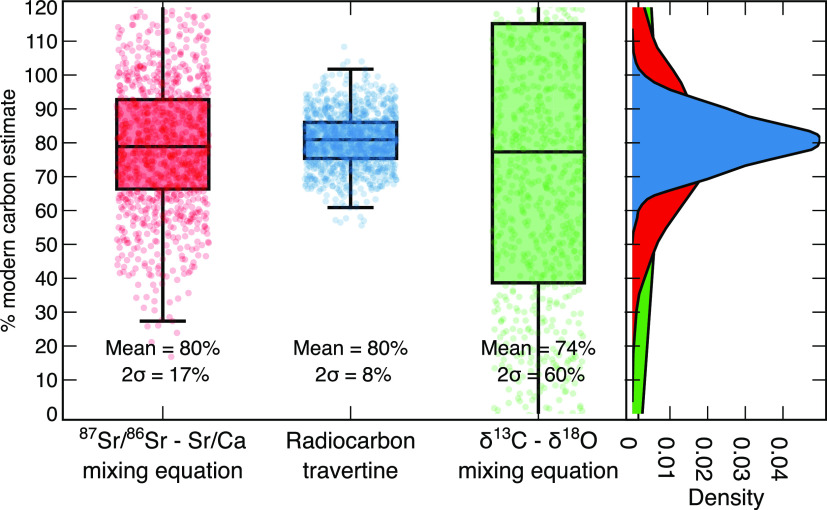
Comparison of estimations of the % of modern carbon being removed at Howden Burn. 2σ uncertainties were propagated using a Monte Carlo routine with 1000 permutations. Means and 2σ uncertainties are presented beneath each box plot.

Radiocarbon data suggest that the proportion of modern carbon in authigenic CaCO_3_ at Howden Burn is ∼80% (2σ = 8%), which agrees with ^87^Sr/^86^Sr–Sr/Ca provenance tracing data (80%, 2σ = 17%) and δ^13^C and δ^18^O data (74%, 2σ = 60%). Radiocarbon provides the most robust constraint on the carbon source once uncertainties are propagated (Figure 4), whereas δ^13^C and δ^18^O data are the least robust, with large uncertainties (2σ = 60%, Figure 4). Using ^87^Sr/^86^Sr and Sr/Ca as provenance tracers provides a reasonable assessment of carbon sources (Figure 4). At enhanced weathering sites where CO_2_ is not being directly mineralized (i.e., remaining dissolved in solution), radiocarbon measurements of waters promise the same unique solution.

### Sources of Carbon Contributing to DIC at Howden Burn

Approximately 20% of the carbon being sequestered in authigenic CaCO_3_ at Howden Burn is not from the modern atmosphere (Figure 4), which suggests that a geological source of carbon with depleted ^14^C is contributing to the carbon budget at Howden Burn. As previously discussed, geological sources of carbon are likely from weathering of local geology (Pennsylvanian coal bearing clastic sediments) or oxidation of OC_petro_ in soils. Radiocarbon measurements of soil overlying the slag deposit have a mean pMC value of 40% (Table 2). Soil organic carbon typically has a modern pMC value,^41^ whereas OC_petro_ will be entirely depleted in ^14^C, i.e., pMC = 0%. Soil radiocarbon data suggest that the carbon pool in soils at Consett is a mixture between modern labile organic carbon and OC_petro_. OC_petro_ can be oxidized to produce aqueous CO_2_, and recent work suggests that this can be a significant source of acidity for chemical weathering,^19^ dependent on O_2_ availability^42^ and temperature.^19^ However, outside specific examples,^18,19^ coal and coke are not known to be significant sources of labile carbon. Carbon supplied by soils is likely to be dominated by the biological respiration of labile organic matter, as opposed to OC_petro_. Future studies should study the radiocarbon composition of soil pore waters to partition labile organic carbon and OC_petro_.^43^

Without a detailed understanding of the rate of oxidative weathering of OC_petro_ and the partitioning between carbonate and silicate weathering in spring water, it is not possible to discern the DIC contributions of these two sources of geological carbon. However, this demonstrates an important nuance for MRV at enhanced weathering sites with regards to additional sources of DIC, as seasonal variations in rainfall (SI Figure 2) and temperature^19^ could influence the contribution of geological carbon to DIC budgets, hence radiocarbon measurements and carbon removal estimates.

For example, the rate of carbonate weathering increases as a function of rainfall.^44−46^ If MRV of an enhanced weathering site was conducted during wetter months (e.g., October to March at Consett, SI Figure 2), alkalinity contributions from carbonate weathering may constitute a larger proportion of the carbon budget. Without thorough radiocarbon-based carbon source apportionment, this would artificially inflate carbon removal rates, as carbonate weathering contributes a mix of modern and geological carbon to stream waters. Similarly, O_2_ availability and temperature controls the rate of both OC_petro_ oxidation and biological respiration of labile organic matter,^19,42^ which mediate the acid hydrolysis of slag material (e.g., eq 2). Given these controls, during drier months the effects of OC_petro_ oxidation on DIC at Howden Burn would be greatest and suppressed during winter months, highlighting the need for reliable carbon source apportionment methods, such as that outlined in this study.

Here, we rely on authigenic CaCO_3_ to provide a time integrated assessment of the proportion of modern carbon being sequestered; in typical enhanced weathering settings, pedogenic carbonates could be used for this purpose.^47^ However, it is not often the case that pedogenic carbon is observed as a consequence of ERW.^14^ In this instance, the DIC content of stream waters would provide the same unique solution, because radiocarbon measurements can be corrected for fractionation effects.^38^ The ability to partition sources of carbon derived from ERW sites in both mineralized and aqueous form makes radiocarbon measurements a versatile tool for quantifying CO_2_ removal rates.

### Developing a Mechanistic Understanding of Weathering Reactions at Enhanced Weathering Sites

Borehole water chemistry can provide useful insights into the dissolution reactions occurring within the slag deposit. Previous studies have suggested that the dominant dissolution reactions at Consett follow eq 1,^24^ or a mixture of eq 1 and eq 2.^26^ Others suggest, based on slag composition data, that the reactions are principally between aqueous CO_2_ derived from the recent degradation of organic matter and silicate phases (e.g., eqs 2 and 4).^22^

Water chemistry aids the development of a mechanistic understanding of dissolution reactions at Consett. Here, we assume that the borehole water chemistry is representative of the dissolution reactions occurring in the slag deposit. If eq 2 is the dominant dissolution mechanism, then reaction stoichiometry dictates that the aqueous Ca/Si ratio (Mol/Mol) is ∼2, whereas if eq 1 is the dominant dissolution mechanism, then the aqueous Ca/Si ratio should be much greater, unless there is a previously unacknowledged sink for Si (e.g., clay minerals). Since the measured Ca/Si ratio in the borehole is 42, we suggest that either (i) eq 1 is the dominant dissolution mechanism, with eq 2 playing a more minor role, or (ii) there is a clay sink, removing Si from waters. We contend that a Si sink in clays is an unlikely explanation for high Ca/Si ratios in borehole and water samples. Although trace amounts of montmorillonite were found in XRD surveys of authigenic CaCO_3_ samples, Si data suggest that montmorillonite is not acting as a quantitative sink of Si at Howden Burn. Si concentrations remain invariant from the borehole to the mouth of Howden Burn, and stream data show consistent Si/Cl ratios (Table 1). This suggests that Si is not quantitatively impacted by authigenic clay precipitation. Montmorillonite may be detrital in origin, as smectite-type clays occur commonly in UK soils.^48^ This evidence suggests that Si is behaving conservatively in this system as suggested previously,^49^ making it likely that the rapid dissolution kinetics of portlandite, relative to silicate phases, explains the high aqueous Ca/Si ratios and pH of waters at Howden Burn.

**Figure 5:**
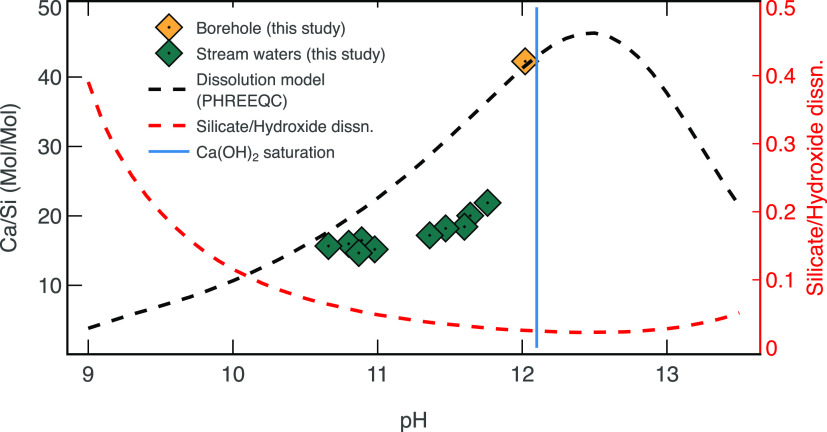
Equilibrium dissolution model for gehlenite, Ca_2_Al_2_SiO_7_; akermanite, Ca_2_MgSi_2_O_7_; and portlandite, Ca(OH)_2_ (black line), and the proportion of silicate/hydroxide dissolution (red line) at a range of pH values. Borehole data are satisfied by a silicate/hydroxide dissolution ratio of 0.025. Stream waters deviate from borehole chemistry due to both carbonate precipitation and mixing. The pH of a solution saturated by Ca(OH)_2_ is shown (blue line) given a *K*_sp_ of 7.9 × 10^–6^.

To quantify the partitioning between hydroxide and silicate dissolution, an equilibrium dissolution model was developed using PHREEQC v3 (lnll database, Figure 5).^50,51^ We assume the dissolving minerals to be gehlenite, akermanite, and portlandite.^22^

Ca/Si ratios and pH in the borehole can be best explained by the majority of dissolution being derived from hydroxide phases (Figure 5). The silicate/hydroxide dissolution proportion suggested by modeling in this study is 0.025 (molar ratio), meaning that eq 1 and eq 3 are likely to dominate carbon removal at Howden Burn. Stream water data deviate from modeled water chemistry as a consequence of both minor mixing with spring water sources and CaCO_3_ precipitation, which removes Ca from solution (Figure 5). Interestingly, the pH of borehole waters is also proximal to the pH of a solution saturated with Ca(OH)_2_. Tentatively, we suggest that the rapid leaching of portlandite in the slag deposit is the reason for declining saturation states in long-term water chemistry, prior to extensive ground works (1978 to 2000) at Howden Burn and the nearby Dene Burn, corroborating previous work.^26^

Our simple model suggests that the low temperature alteration of steel slag is dominated by hydroxide dissolution within the slag deposit at Consett, with a minor chemical contribution from silicate dissolution (<3%, Figure 5). This is at odds with laboratory experiments, which determine rates of slag carbonation—often achieving congruent dissolution of steel slags.^52,53^ It is likely that reaction conditions in laboratory experiments and the Consett site are not comparable.^26^ Experimental studies often use higher liquid/solid ratios (e.g., 0.4),^52^ CO_2_ partial pressures (e.g., 1 to 30 bar),^53^ and temperatures (e.g., 20 to 200 °C)^53^ than would be experienced within the slag deposit at Consett. Furthermore, some experiments appear to exhaust Ca(OH)_2_,^53^ and others have none present.^52^ Consequently, the rates of carbonation derived during laboratory experiments may not be comparable to the carbonation rates of steel slags in an open natural system. This highlights the importance of transitioning enhanced weathering experiments from laboratories to natural systems.

### Quantifying CO_2_ Removal Rates Using Radiocarbon Measurements

Quantifying the rate of carbon removal from an enhanced weathering and mineralization site relies on knowledge of the main reactions contributing solutes to the dissolved load (at Howden Burn, this is eq 1), carbon source apportionment (provided by radiocarbon data in this study), and the fraction of DIC export to the oceans. This latter point is comparing the amount of direct mineralization of CO_2_ on land and the amount of CO_2_ stored as alkalinity in the oceans (i.e., ocean alkalinity enhancement). This is important to quantify because direct mineralization results in 1 mol of CO_2_ removal per mole of CaCO_3_ precipitated, whereas ocean alkalinity enhancement results in 1.4 to 1.7 mol of CO_2_ removal per mole of mineral dissolved, once carbon speciation effects as a result of ocean salinity, temperature, and pH have been accounted for.^28^ Here, we assume that carbon leaving Howden Burn’s catchment will be transported to the ocean. The rate of carbon removal (κ, t C y^–1^) is calculated by
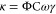
5γ is the fraction of modern carbon, calculated from radiocarbon data (0.8, Figure 4). ΦC is the total carbon flux at Howden Burn (t C y^–1^), and ω is the uptake efficiency of CO_2_, which is a function of the fraction of carbon exported to the oceans vs directly mineralized on land. ω is calculated as follows:
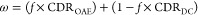
6*f* is the fraction of carbon exported to the oceans (i.e., 1 – fraction of alkalinity lost between BHW1 and W01), CDR_OAE_ is the CO_2_ removal efficiency of ocean alkalinity enhancement (1.55), and CDR_DC_ is CO_2_ removal efficiency of direct carbonation on land (1).^28^ Here, we assume that the monotonic decrease in alkalinity and [Ca+Mg] downstream (Figure 2a) is forced by CaCO_3_ precipitation rather than dilution, because Howden Burn chemistry does not follow a dilution trend when compared to runoff (SI Figure 4),^44^ and time-series data show that Howden Burn is generally oversaturated with respect to calcite (SI Figure 4). This suggests that carbonate precipitation happens throughout most of the year and is the principal control over river chemistry (Figure 2).

**Figure 6:**
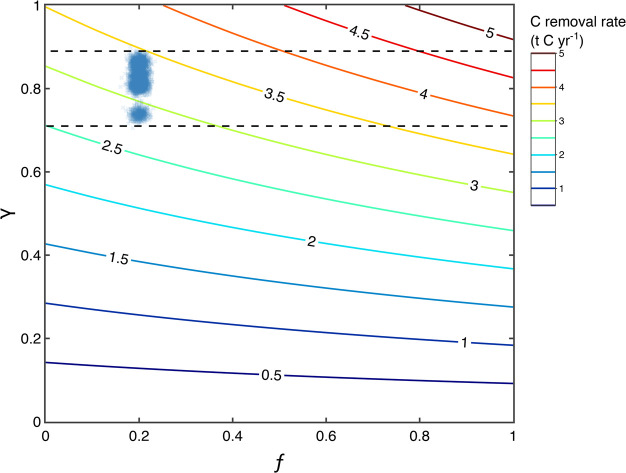
Temporal carbon removal rate (tonnes C y^–1^) at Howden Burn, Consett. Colored contours represent carbon removal rates, derived from eq 6. Blue data are derived from authigenic CaCO_3_ pMC data and alkalinity loss, with uncertainties propagated by the Monte Carlo routine (permutations = 1000). *f* is the fraction of carbon exported to the oceans, and γ is the fraction of carbon that is derived from modern sources, relative to geological. Black dashed lines envelope potential carbon removal rates throughout the year. The full calculation is shown in the SI.

Radiocarbon measurements on authigenic CaCO_3_ presented in this study provide the most accurate estimation of γ to date (0.8, Figure 4). The water chemistry data (collected in July 2021) estimate ω is 1.12, where *f* = 0.2, and long-term chemistry data show that ΦC is 3.8 t y^–1^ (SI). Given these parameters, the rate of carbon removal (κ) at Howden Burn is estimated to be 2.7–3.5 t C y^–1^ by eq 6 (Figure 6), which is within previous estimates (0.8–9.4 t C y^–1^)^22^ and provides a considerable improvement in uncertainty. Seasonality at Howden Burn will induce changes in physical parameters such as river discharge and temperature, which will impact the fraction of modern carbon contributing to alkalinity (as discussed earlier) and also the fraction of DIC exported to the oceans (*f*). Assuming radiocarbon data presented in this study represent a time integrated γ value, and if ω varies during the year as a function of *f*, κ may be between 2.5 and 4.7 t C y^–1^ (black dashed lines, Figure 6). Because the reactivity of steel slags has likely decreased over the past 40 years, as no new material has been placed in that time, these C removal rates are likely less than the maximum potential.

An assumption inherent in eq 6 is that the fraction of alkalinity not precipitated within the Howden Burn catchment reaches the oceans. Given the Derwent’s current chemical composition (SI Table 1), the potential transport capacity of carbon^5^ suggests the Derwent can transport an additional 500 μmol L^–1^ of alkalinity charge balanced by Ca^2+^, before reaching oversaturation with respect to calcite. This equates to an additional 4800 t C y^–1^ that can be exported by Derwent to the much larger River Tyne. Normalized to catchment area (350 ha),^26^ this is equal to 14 t C ha^–1^ y^–1^. The mean flow of the Derwent at a sampling site proximal to Howden Burn at Rowlands Gill is 2.6 × 10^3^ L s^–1^ (data from https://nrfa.ceh.ac.uk/data/station/meanflow/23007). The mean flow estimated at Howden Burn is 6 L s^–1^,^26^ and the alkalinity at the mouth of Howden Burn was 864 μmol L^–1^ at the time of sampling (Table 1, Figure 2a). Therefore, the addition of alkalinity to Derwent from Howden Burn is estimated to increase Derwent’s alkalinity from ∼754 μmol L^–1^ to ∼756 μmol L^–1^, contributing a negligible amount of additional alkalinity to Derwent, in comparison to the amount of alkalinity Derwent could carry prior to becoming oversaturated with respect to calcite—1250 μmol L^–1^. The potential transport capacity of carbon calculated here is inorganic and does not account for the biological uptake of alkalinity, which may reduce the transport capacity further.

## Implications

As researching enhanced rock weathering for CO_2_ removal transitions from mesocosm experiments to much larger field scale trials, the need for robust methods to quantify CO_2_ removal rates will be pressing. This study provides an initial attempt at comparing different geochemical methods for quantifying CO_2_ removal rates and couples geochemical measurements to geochemical modeling to gain a deeper understanding of dissolution and precipitation reactions at a well-established enhanced weathering site in Consett, Co. Durham. We find that using radiocarbon measurements of calcite precipitates is the method resulting in the lowest uncertainty for disentangling the sources of carbon contributing to alkalinity, which predicts that 80% (2σ = 8%) of carbon mineralized at Howden Burn is modern. We use provenance tracers (e.g., ^87^Sr/^86^Sr) to show that mixing from other water sources is a negligible process at the site. Equilibrium geochemical dissolution models are used to understand the water chemistry at the draining site, though this relies on the chemistry of the solid undergoing dissolution being well characterized. The amount of carbon exported to the ocean can then also be calculated as a function of mixing (i.e., dilution) and alkalinity loss. Our study determines that current carbon removal rates at Howden Burn, Consett are between 2.7 and 3.5 t C y^–1^, with a potential maximum of ∼5 t C y^–1^.

Although rigorous, radiocarbon measurements are not trivial to make and can be expensive, it is important to emphasize that MRV at scale needs to be both accurate and cost-effective.^13^ Monitoring alkalinity and conductivity are very convenient and cost-effective^13^ but do not partition carbon sources, whereas the methodology outlined here could be expensive at scale and is not as convenient but gives a rigorous assessment of carbon sources. At any ERW site, a balance between more frequently used low-cost and convenient measurements and less frequently used higher effort/cost methods needs to be established, as one without the other would not be satisfactory.

## Supporting Information Available

Extended methods pertaining to particulars of radiocarbon analysis; photographic evidence of carbonate precipitation smothering the river bed at Howden Burn; effluent discharge vs time plot for Rowland’s Gill; XRD spectra confirming calcite mineralogy; long-term saturation index data and runoff for Howden Burn; a derivation of eqs 5 and 6 in the main manuscript, including long-term pH data; a data table detailing water chemistry for the River Derwent, which Howden Burn drains into (PDF)
