# Supplementary material for: Quantifying CO2 Removal at Enhanced Weathering Sites: a Multiproxy Approach
Source: Environ Sci Technol. 2023 Jun 21;57(26):9854–64. doi: 10.1021/acs.est.3c03757 (PMC10324310; doi:10.1021/acs.est.3c03757)
Supplement: Supplementary file 1 — es3c03757_si_001.pdf [file es3c03757_si_001.pdf]

Quantifying CO_2 removal at enhanced weathering sites: a multi-proxy approach

William J. Knapp, Emily I. Stevenson, Phil Renforth, Philippa L. Ascough, Alasdair. C. G. Knight, Luke Bridgestock, Michael J. Bickle, Yongjie Lin, Alex L. Riley, William M. Mayes, Edward T. Tipper

May 10, 2023

Pages = 9, Figures = 5, Tables = 1

Supporting information: Contains extended methods pertaining to particulars of radiocarbon analysis. Photographic evidence of carbonate precipitation smothering the river bed at Howden Burn. Effluent discharge vs time plot for Rowland's Gill. XRD spectra confirming calcite mineralogy. Long-term saturation index data and runoff for Howden Burn. A derivation of Eqn. 5 and 6 in the main manuscript, including long-term pH data. A data table detailing water chemistry for the River Derwent, which Howden Burn drains into.

1 Extended methods

1.1 Radiocarbon analyses

Authigenic $CaCO_3$ samples were pretreated by first removing the outer 20% by weight of the sample, to remove surface contaminants, by etching with weak HCl under vacuum in a gas-tight exetainer. Evolved CO_2 was removed, and the remaining pretreated sample was then converted to CO_2 by complete hydrolysis using 2N HCl. Soil samples were pretreated by acid fumigation. The sample was moistened with a small amount of deionised water, covered by glass fibre filter and placed into a glass vessel together with a beaker of concentrated HCl to hydrolyse and carbonate in the sample over three days. The total carbon in a known weight of the pre-treated sample was recovered as CO_2 by heating with CuO in a sealed quartz tube. CO_2 from both authigenic $CaCO_3$ and soil samples was cryogenically purified on a vacuum line. An aliquot of the sample CO_2 was then taken for off-line δ^{13}C measurement on a ThermoFisher Delta V stable isotope ratio mass spectrometer. This δ^{13}C value was then used for normalisation of the measured $^{14}C/^{13}C$ ratiosStuiver and Polach (1977). A second aliquot of purified sample CO_2 was taken for conversion to graphite by the Fe/Zn reduction method (Slota et al., 1987). Prepared graphite was then pressed into pre-drilled Al targets and $^{14}C/^{13}C$ ratios were measured using an accelerator mass spectrometer (AMS) at the SUERC AMS laboratory. Samples were measured to 3‰ uncertainty on AMS. Replicate measurements of sample CON21-T03 were indistinguishable on the basis of a χ^2 test (df = 1, n = 2) = 0.08, p <0.1.

2 Carbonate smothering at Howden Burn

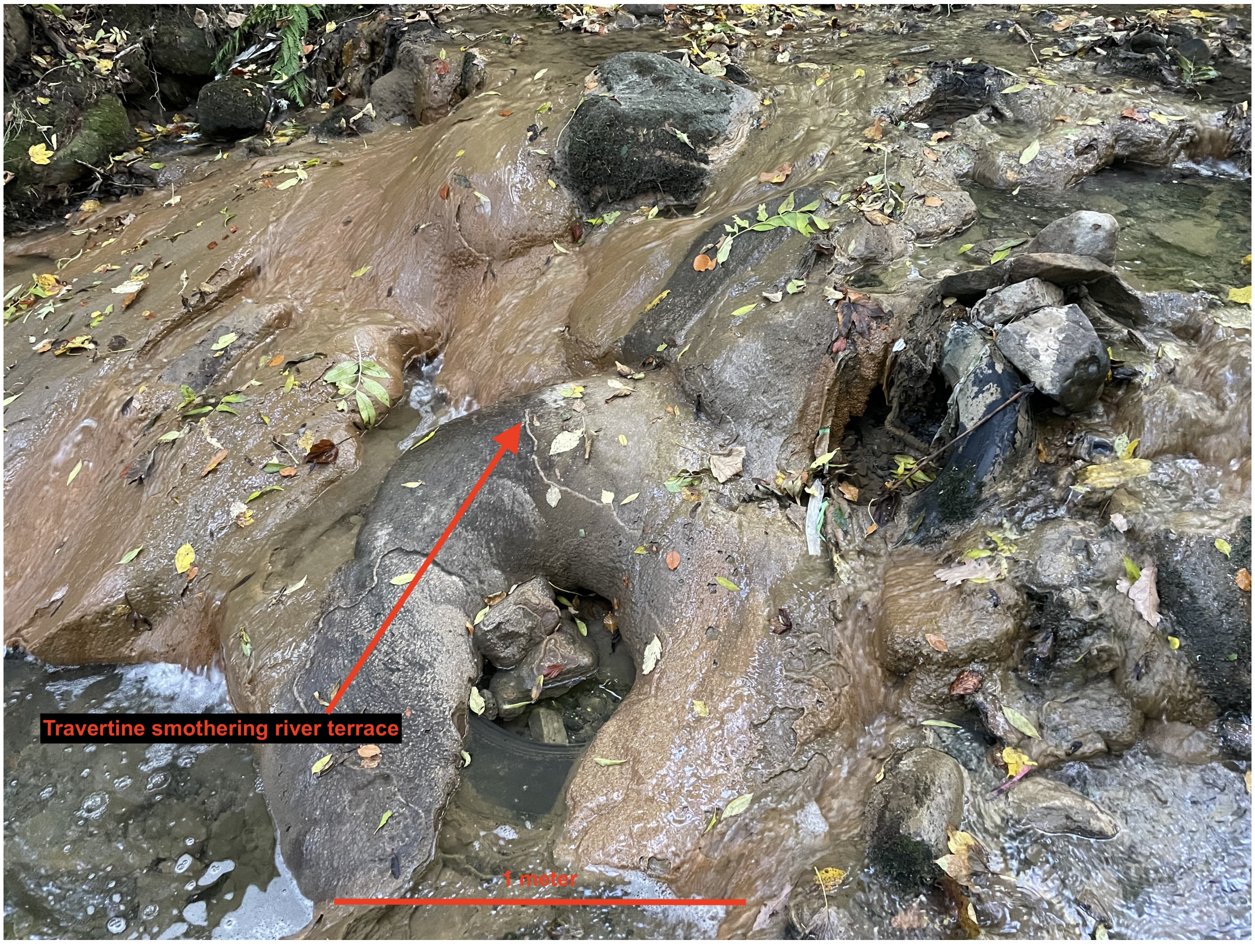

Figure 1: Travertine smothering a river terrace at Howden Burn.

3 Hydrological conditions at Rowland's Gill

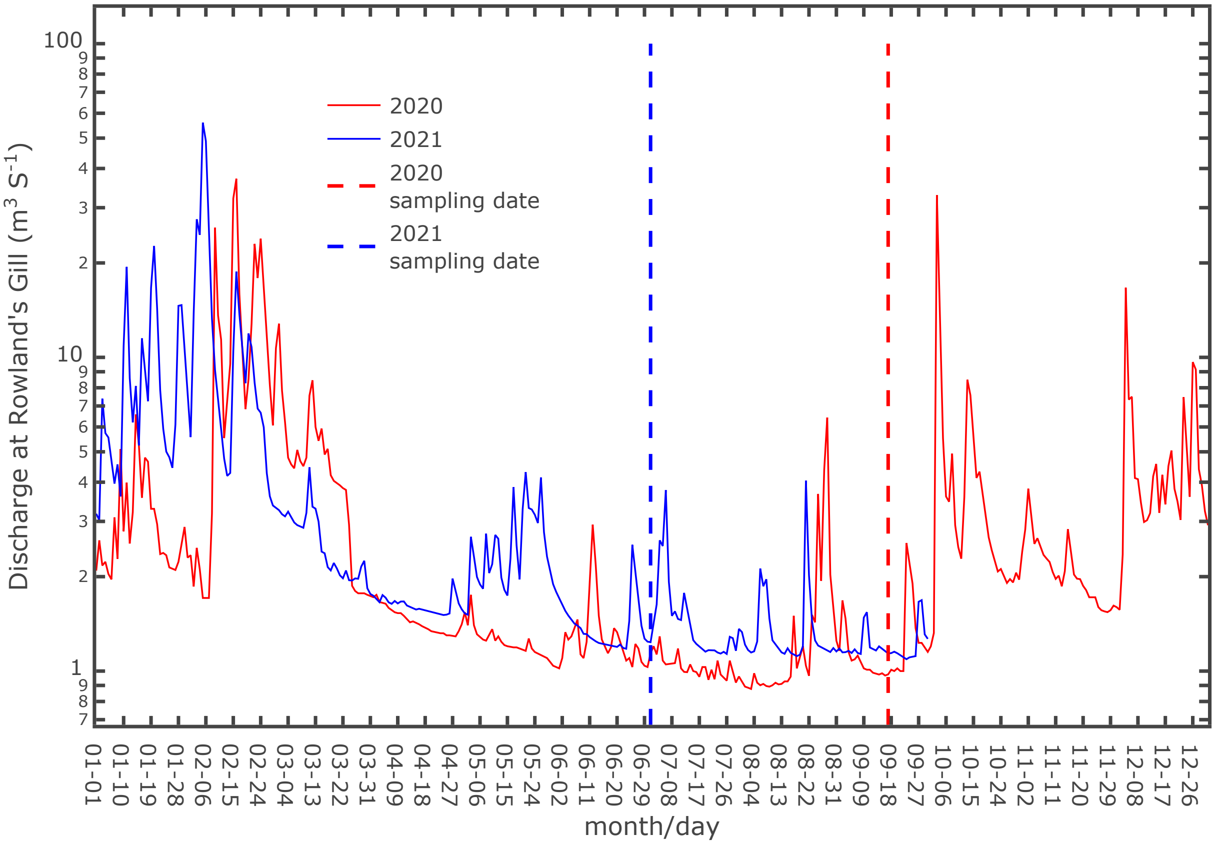

Figure 2: Discharge data from Rowland's Gill (Derwent), a gauging station downstream of Howden Burn. Discharge data are shown for years 2020 and 2021, when water and travertine samples with code CON20 and CON21 were collected. Discharge data are available via National Rivers Flow Archive: https://nrfa.ceh.ac.uk/data/station/meanflow/23007

4 XRD analysis

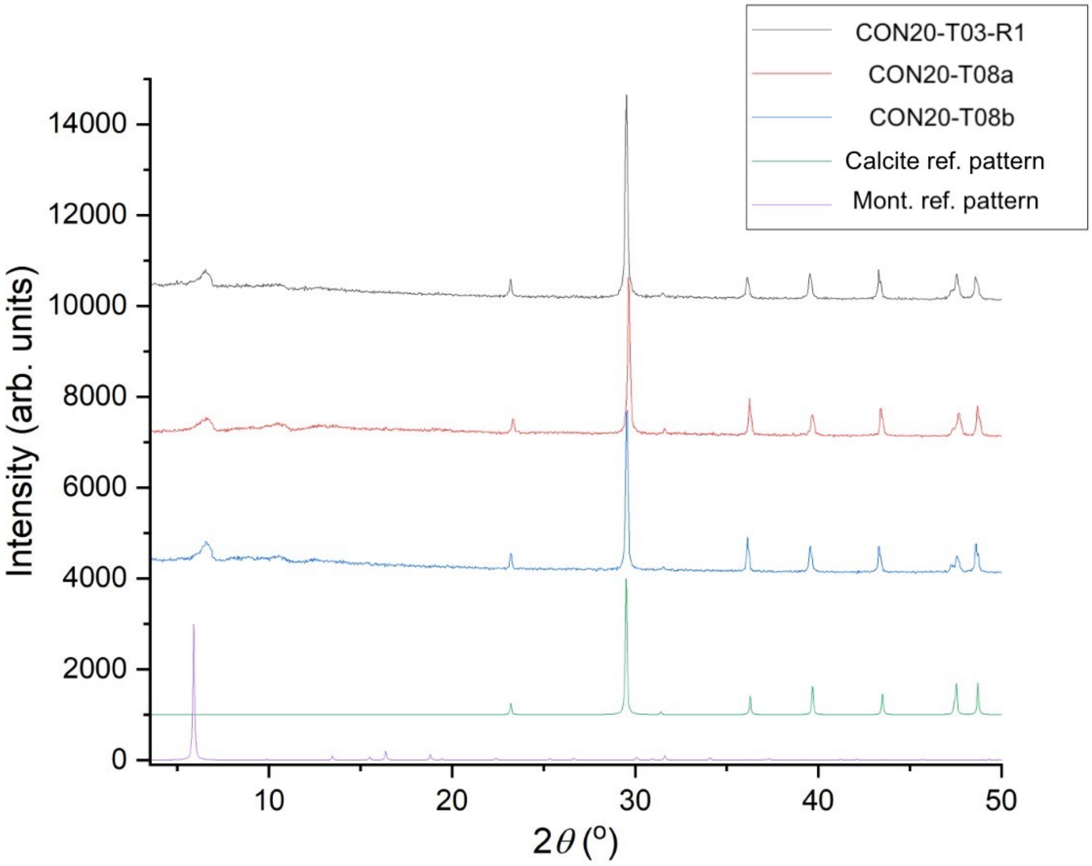

Figure 3: XRD data for travertine samples, with reference patterns for calcite and montmorillonite. reference patterns displayed in the figure were calculated from structural models from the Inorganic Crystal Structure Database (reference codes 161171 and 40109 for montmorillonite and calcite respectively after Allmann and Hinek, 2007).

5 Source to sink carbon quantification Howden Burn

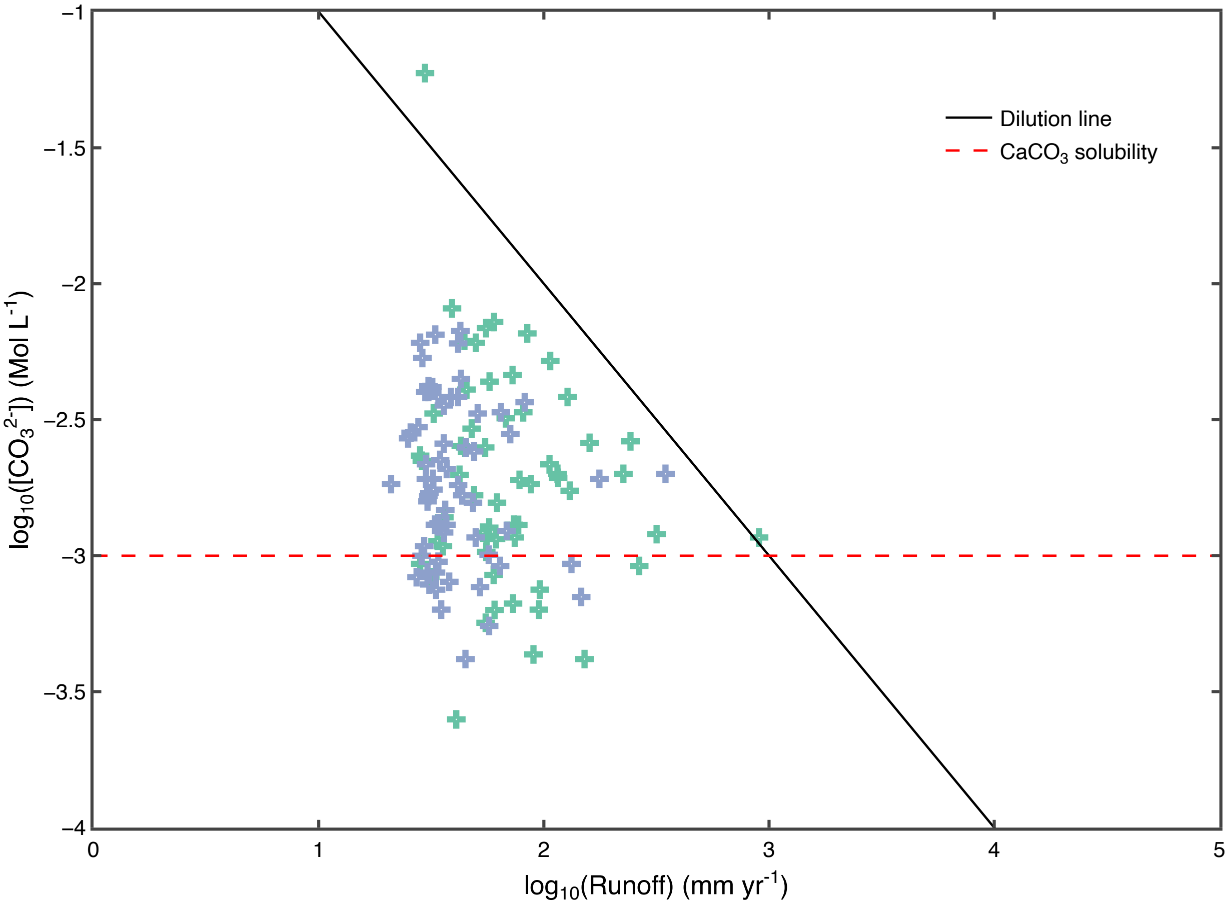

Figure 4: Long term chemical and runoff data from Howden Burn, spanning from 1978 - 2014. Blue points represent summer measurements, green points represent winter measurements. A dilution trend, negative unity, is shown (black line). Chemical data do not follow the expected dilution trend. Calcite solubility is also shown (red line), for a solution of pH 11 and 10 °C. The majority of chemical measurements shown are oversaturated with respect to calcite.

Derivation of equation 4

The rate of carbon removal (κ, t C yr^{-1}) is defined by:

$$\kappa = \Phi C \omega \gamma \tag{1}$$

Where γ is the fraction of modern carbon, calculated from radiocarbon data, and ω is the fraction of carbon exported to the oceans.

ΦC is the total C flux (t yr^{-1}), and this is calculated as the product of the mean alkalinity flux from the long term data set kindly provided by William Mayes and the atomic mass of C. The mean molar value is 0.01 Mol s^{-1}, which has a variance of 0.001 Mol s^{-1} and a 2σ uncertainty of 0.06 Mol s^{-1}. The large uncertainty on this parameter is likely a result of various physical interventions employed at the site during the last 40 years, which resulted in changes in dominant flow paths within the slag heap. This may be seen in long term pH records, which show marked decreases in pH at Howden Burn for 15 years following the site closure (red circles, Fig. 5). The total mass flux of carbon per year at Howden Burn is equal to the product of the molar alkalinity flux, amount of seconds in a year, atomic mass of HCO_3^- and the molar fraction of C in alkalinity (20%). An alkalinity flux of 0.01 Mol s^{-1} is therefore equal to a carbon flux (ΦC) of 3.8 t C yr^{-1}, before correction.

ω is calculated using downstream alkalinity measurements, which are assumed to be solely influenced by carbonate precipitation reactions (SI Fig: 4). Alkalinity decreases from 5160 μMol/L to 864 μMol/L from source to mouth at Howden Burn, which means $\sim$ 20 % of carbon is exported to the ocean. A mixing equation (Eqn.6 in main text) can then be used to calculate ω, with values for carbon removal efficiencies by direct carbonation vs ocean export from Renforth and Henderson (2017):

$$\omega = (f \times CDR_{OAE}) + (1 - f \times CDR_{DC}), \quad (2)$$

where f is the fraction of alkalinity exported to the ocean, CDR_{OAE} is CO_2 removal efficiency of ocean alkalinity enhancement (1.55) (Renforth and Henderson, 2017), and CDR_{DC} is the CO_2 removal efficiency of direct carbonation on land (1).

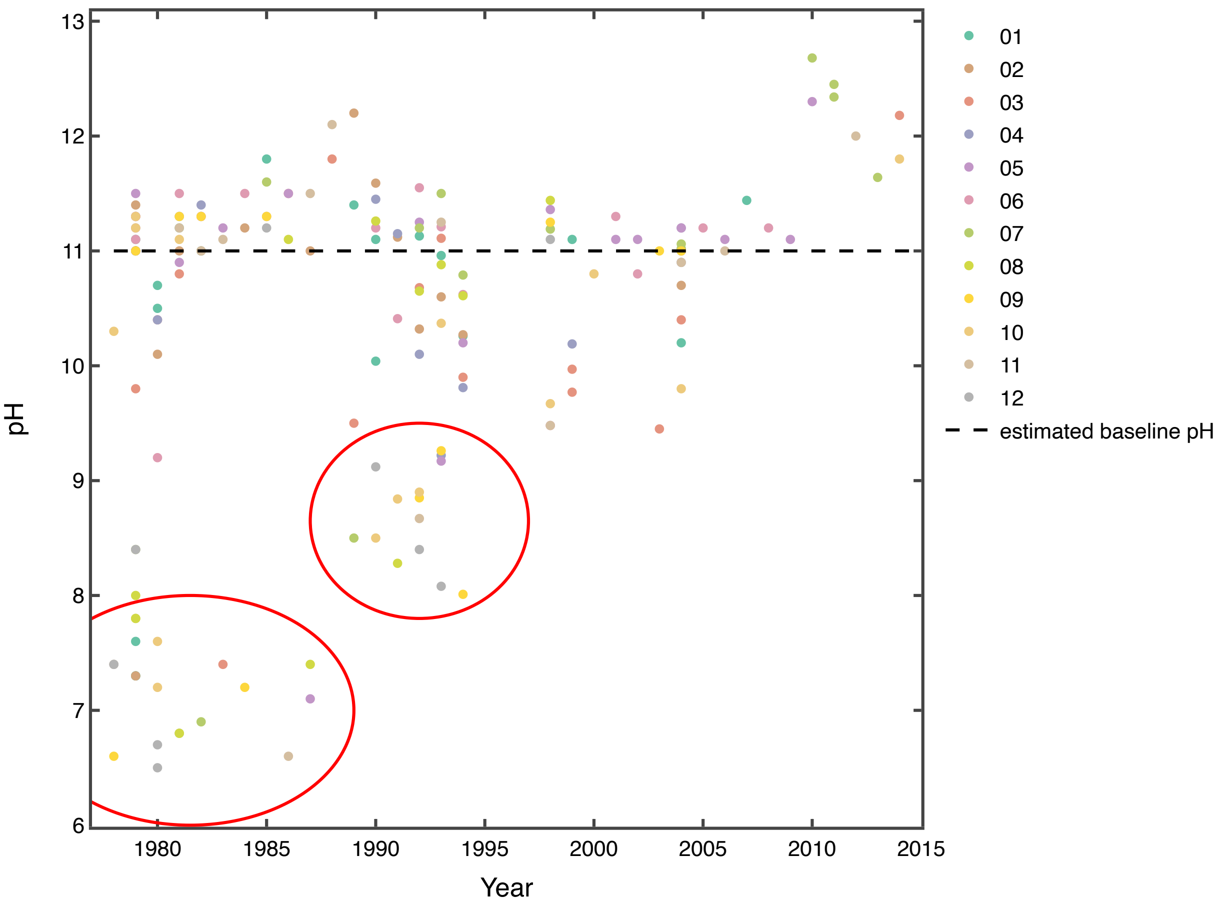

Figure 5: Temporal pH record at Howden Burn. Red circles show hypothesised flow-path change events at the site (red circles). Points are coloured by month of the year

6 River chemistry, Derwent

Sample ID	long	lat	pH	Alkalinity	Ca	Mg	Na	S	Si	Sr
Derwent_W01	54.85	-1.87	6.82	754.76	381.74	148.12	556.77	149.70	60.53	0.80

Table 1: Chemical composition of Derwent river water, sampled during this study. Chemical concentrations are reported in μMol/L

References

Allmann, R., and R. Hinek, 2007: The introduction of structure types into the inorganic crystal structure database icsd. *Acta Crystallographica Section A: Foundations of Crystallography*, **63 (5)**, 412–417.

Renforth, P., and G. Henderson, 2017: Assessing ocean alkalinity for carbon sequestration. *Reviews of Geophysics*, **55 (3)**, 636–674.

Slota, P., A. T. Jull, T. Linick, and L. Toolin, 1987: Preparation of small samples for 14C accelerator targets by catalytic reduction of CO. *Radiocarbon*, **29 (2)**, 303–306.

Stuiver, M., and H. A. Polach, 1977: Discussion reporting of 14c data. *Radiocarbon*, **19 (3)**, 355–363.
